# Mesenchymal stem/stromal cells—a key mediator for regeneration after perinatal morbidity?

**DOI:** 10.1186/s40348-016-0034-x

**Published:** 2016-02-11

**Authors:** Martin Mueller, Tim G. A. Wolfs, Andreina Schoeberlein, Antonio W. D. Gavilanes, Daniel Surbek, Boris W. Kramer

**Affiliations:** Department of Obstetrics, Gynecology, and Reproductive Sciences, Yale University School of Medicine, New Haven, CT USA; Department of Obstetrics and Gynecology, University Hospital Bern and Department of Clinical Research, University of Bern, Bern, Switzerland; Department of Pediatrics, Maastricht University Medical Center (MUMC), Maastricht, The Netherlands; School of Oncology and Developmental Biology (GROW), Maastricht University, Maastricht, The Netherlands; Institute of Biomedicine, Facultad de Ciencias Médicas, Universidad Católica de Santiago de Guayaquil, Guayaquil, Ecuador; Department of Neuropsychology, Division Neuroscience, School of Mental Health and neuroscience (MHeNS), Maastricht University, Maastricht, The Netherlands

**Keywords:** Perinatal brain injury, Hypoxic-ischemic encephalopathy, Bronchopulmonary dysplasia, Mesenchymal stem cells, Neonatal, Perinatal

## Abstract

Perinatal complications in both term- and preterm-born infants are a leading cause of neonatal morbidities and mortality. Infants face different challenges in the neonatal intensive care unit with long-term morbidities such as perinatal brain injury and bronchopulmonary dysplasia being particularly devastating. While advances in perinatal medicine have improved our understanding of the pathogenesis, effective therapies to prevent and/or reduce the severity of these disorders are still lacking. The potential of mesenchymal stem/stromal cell (MSC) therapy has emerged during the last two decades, and an increasing effort is conducted to address brain- and lung-related morbidities in neonates at risk. Various studies support the notion that MSCs have protective effects. MSCs are an easy source and may be readily available after birth in a clinical setting. MSCs’ mechanisms of action are diverse, including migration and homing, release of growth factors and immunomodulation, and the potential to replace injured cells. Here, we review the pathophysiology of perinatally acquired brain and lung injuries and focus on MSCs as potential candidates for therapeutic strategies summarizing preclinical and clinical evidence.

## Introduction

Despite advances in obstetrics and neonatology, both preterm- and term-born infants continue to face serious risks during pregnancy, parturition, and adaptation after birth. Perinatal medicine is therefore a major public health issue around the world. The clinical presentation after perinatal complications or preterm birth in an individual child is complex. This complexity results from multiple potential causal pathways, signs, and symptoms of injury. Typical pathologies in these newborns include perinatal brain injury and bronchopulmonary dysplasia (BPD), among others. New avenues to treat these morbidities have emerged among which stem cells being particularly promising. The body of knowledge of stem cell biology and their function in tissue regeneration and protection has broadened exponentially in the last two decades. This review will discuss mesenchymal stem/stromal cells (MSCs) as a therapeutic approach for both brain and lung diseases in infants at risk. Furthermore, we will highlight the preclinical and clinical data that have emerged on the role of MSCs in perinatal medicine.

## Perinatally acquired organ injury

Perinatally acquired organ injury affects both term and preterm infants. Depending on the timing of injury and/or delivery, infants need to cope with different challenges. In the developing brain of a preterm infant, the spectrum of injury suggests that the underlying pathophysiology is not due to a single lesion but consists of white and gray matter disturbances [1]. Thus, a comprehensive multidimensional assessment of potential contributing factors such as maternal medical history, obstetric antecedents, intrapartum factors (including fetal heart rate monitoring results and issues related to the delivery itself), and placental pathology is recommended [2]. In the term-born infants, perinatal insults such as birth asphyxia or perinatal stroke affect 1 to 3 newborns out of 1000 [3, 4]. In contrast, in preterm-born infants morbidity and mortality strongly relate to the gestational age. While preterm birth before 37 weeks of gestation occurs in 5–8 % of all pregnancies, very low gestational age (VLGA) before 32 weeks of gestation occurs in about 1 % of singletons and 9 % of twin pregnancies [5]. Mortality of VLGA infants ranges between 7.3 and 21.4 % at 30 days and 9.0 and 22.7 % at 1 year [6]. Additionally, a large number of survivors suffer significant long-term disabilities including cerebral palsy (CP), epilepsy, increased hyperactivity, and developmental disorders [7]. For example, the risk to develop CP is 30 times higher in infants born before 33 weeks of gestation compared to term-born infants [8]. Moreover, injury in these infants is frequently exacerbated by fetal inflammation and preferentially affects cerebral white matter resulting in periventricular leukomalacia and germinal matrix hemorrhage [1]. Currently, the only intervention known to reduce the burden of perinatal brain injury in the term population is hypothermia. Several large clinical trials confirmed that hypothermia in infants with neonatal hypoxic-ischemic encephalopathy is associated with a significant reduction in death and disability [9]. However, 40–50 % of infants treated with hypothermia still die or develop significant neurological disability [10]. In the preterm population, therapeutic options are lacking as hypothermia is contra-productive. Antenatal magnesium sulfate prior to birth at less than 30 weeks of gestation reduces CP and combined CP and mortality rate at 2 years of age. However, randomized control trials do not demonstrate long-term neurological benefits [11, 12]. Approximately 25 % of VLGA infants will develop BPD with long-lasting consequences such as chronic respiratory impairment and neurodevelopmental delay [13]. Changes in clinical management reduced the incidence of BPD significantly with a shift from VLGA to extreme low gestational age newborns developing BPD. Not surprisingly, the current pathogenesis of BPD is based on immaturity with disordered alveolar and capillary development and represents a developmental disorder [14]. The plasticity of the developing lung after preterm birth is poorly understood. Long-term follow-up studies suggest an incomplete regeneration of the lung growth in survivors with BPD. Current treatment options for BPD include vitamin A and caffeine in addition to supportive therapies, but their results often remain unsatisfactory [15].

Together, therapeutic approaches to counteract the consequences of perinatally acquired injury are sparse, and new measures are desperately needed especially for preterm infants [1, 15–17].

## Pathophysiology of brain and lung injury

Experimental studies identified different phases of perinatally induced neuronal death. Primary neuronal death is related to depletion of tissue energy reserves with primary energy failure. The secondary and tertiary phases are related to excitotoxicity, mitochondrial dysfunction, and free radical accumulation leading to cell necrosis or apoptosis with impaired myelination and/or axonal function [18]. The secondary and tertiary phases may cause persistent inflammation and gliosis, sensitization to further injury, and impaired oligodendrocyte maturation and myelination [19]. Interestingly, inflammation is increasingly recognized as being a critical contributor to both normal development and injury outcome especially in the immature brain [20]. Maternal infection/inflammation is not only a major risk for preterm birth but is linked to systemic fetal inflammatory response which, in turn, may elicit injury in the fetus. Perinatal inflammation modulates vulnerability to and development of brain injury [21] and influences critical phases of myelination and cortical plasticity [20]. Several studies suggest that inflammation may play a critical role in autism and schizophrenia [22]. Together, brain development, myelination, vascularization, and apoptosis are strongly influenced by inflammatory responses in both physiologic and pathophysiologic conditions [20, 23]. Pivotal regulators of inflammatory responses in the brain are glial cells which orchestrate the release of pro- and anti-inflammatory cytokines [24, 25]. Pro-inflammatory cytokines such as tumor necrosis factor (TNF)-α and interferon gamma (IFN-γ) are cytotoxic to oligodendrocytes [26]. However, glial cells may produce anti-inflammatory cytokines (e.g., IL-10), which suppress expansion of IL-1β and TNF-α and contribute to resolving inflammation and repair processes [25]. Further, astrogliotic scar formation installs a barrier around tissue lesions to restrict the crossing of inflammatory cells into surrounding healthy areas [24].

The regulation of the inflammatory responses in the newborn appears to be a link that may explain some of the common features of organ injury in preterm infants. Many studies identified characteristic inflammatory changes and altered growth factor signaling in BPD such as initial influx of neutrophils into the lung followed by increased numbers of macrophages [27]. The release of cytokines and disturbance of growth factor signaling such as transforming growth factor (TGF)-β results in increased apoptotic process [28]. Furthermore, several studies showed that dysmorphic capillaries and subsequent development of pulmonary hypertension are related to an altered pattern of angiogenic growth factors such as vascular endothelial growth factor (VEGF) and its receptors [29]. Finally, lung injury leads to remodeling or early alveolar epithelial dysfunction which in turn promotes lung inflammation [30].

Taken together, perinatal inflammation is a strong modulator of both physiologic and pathophysiologic development of the neonatal organs. The fetal inflammatory response to certain cues such as lipopolysaccharides (LPS) [31] can be detected not only in the brain and the lung but also in remote tissues not directly exposed to LPS such as the spleen, liver, and mediastinal lymph nodes [32–34]. Protective strategies to counteract the cascades leading to injury should therefore not focus on one organ or system but rather treat perinatally acquired injury globally, in which the immune system plays a key role. MSCs possess a regenerative potential. They were shown to modulate innate and adaptive immune responses, to have antiapoptotic effects, to decrease inflammation, and to enhance tissue repair, mostly through the release of paracrine factors [35, 36].

## Mesenchymal stem/stromal cells

Stem cells are broadly defined as cells with self-renewing and differentiation capacity. Although stem cells derived from embryonic tissue were identified first, the clinical use is limited due to ethical concerns and tumorigenic potential [37, 38]. Clinical and animal stem cell-based studies to prevent or repair perinatally acquired injury have emerged during the recent years with MSCs being particularly promising. These cells are considered somatic stem cells as they originate from stem cell niches such as bone marrow (BM), skin, adipose umbilical cord, and placental tissues [39]. MSCs can be isolated from placental membranes and tissues [40, 41], amniotic fluid [42–44], umbilical cord blood [45, 46], and the umbilical cord connective tissues (Wharton’s jelly; WJ) [47, 48]. Although all of these cells are MSCs, specific considerations with respect to clinical use, time of application, application route, availability, and ethical aspects need to be made.

MSC-based therapies are an attractive strategy since the pathophysiology of perinatally acquired injury is heterogeneous and MSCs have the capacity to adapt to the microenvironment of injured organs. The strategy may be either or both replacement/restoration of lost tissue and/or protection/salvage of injured cells. In term infants at risk for hypoxic-ischemic injury or neonatal ischemic stroke, MSCs could exert a neuroprotective effect starting at the acute phase of injury. The timing and presentation of the injury are usually well defined. MSCs could provide trophic support and/or amelioration of the inflammatory responses, leading to repair or reduced cell death. However, the different cell types, transplantation routes, and the timing need to be accounted for. Given the gold standard therapy of hypothermia for this kind of injury, MSCs have to proof additive/synergistic effects in order to be considered [49]. In contrast, in the preterm population, the timing of the injury is often unclear and the pathophysiology is more complex. The clinical diagnosis of infants at risk is challenging as symptoms such as CP or BPD are diagnosed in early childhood years. Thus, the injury may be considered more chronic as extensive atrophy and gliosis of the white matter tract or dysfunction of lung architecture are present. MSCs could modulate not only the inflammatory response after delivery but also the degree and magnitude of the injured white matter and epithelial cells as well. However, many questions such as altered pattern of growth factors and intercellular matrix proteins that could affect proliferation or differentiation of the desired cell types need to be addressed first.

## MSCs: homing and migration

The approach of MSCs as a therapy for perinatal injury is based on several crucial properties of MSCs, including delivery of the cells “homing” to the site of injury. Migration and homing to the tissue of injury is influenced by multiple factors including age, passage, and number of cells; culture conditions; and delivery method [50]. The apparent migration and homing abilities of MSCs without tumorigenic potential were described by several groups and in different disease models [51–54]. In BPD animal models, peripherally injected cells were detected in the hyperoxia-induced injured lung [55, 56]. Experimental studies identified chemokines as major molecules responsible for cell homing with chemokine receptors CXCR3, CXCR4, and CXCR6 being particularly important [57–59]. Further secretion of factors such as stromal cell-derived factor-1α (SDF-1α), which is a CXCR4 ligand, promotes migration of MSC to the injury site [60]. Interestingly, the phenotype of MSCs is an important criterion as well. CD9 (high)-positive MSCs display improved engraftment compared to the CD9 (low)-positive population in a murine ischemic hind limb model [61]. This observation highlights the rather heterogeneous MSC population and the importance of proper MSC characterization and definition for future studies. Currently, MSC characterization is based on a set of minimal criteria [62], and they display a cell surface repertoire and gene expression pattern which differ among MSCs from various tissues of origin and culture conditions used [63–66]. For example, MSCs derived from amniotic fluid express many cell surface markers characteristic for BM-derived MSCs including CD73, CD90, CD105, and major histocompatibility complex (MHC) class I [44]. The lack of MHC class II, CD40, CD80, and CD86 molecules suggests a low immunogenic phenotype of MSCs when compared to other stem cell sources [67]. In contrast, WJ-MSCs express cell surface markers CD29, CD44, CD73, CD90, CD105, CD146, and CD166 [68, 69] and are considered more primitive cell population relative to BM-derived MSCs [70]. As a result, WJ-MSCs differentiate more efficiently into neural progenitors compared to BM-derived MSC [71]. In addition, the underlying clinical condition may also affect the phenotype of MSCs. WJ-MSCs derived from umbilical cords collected after preeclamptic pregnancies seem to be more committed to neuroglial differentiation compared to cords from uncomplicated pregnancies [66].

## MSCs: secretome and immunomodulation

While MSCs have a proven restorative capacity in response to injury cues, the question of potential protective mechanisms remains unclear. Most of the available data comes from adult neurodegenerative and lung diseases or in vitro studies. Studies identified the induction of cytokines, interleukins, and trophic factors predominately involved in neurogenesis, angiogenesis, hematopoiesis, and cardiovascular regeneration being crucial for the mostly paracrine effects [58, 72]. For example, WJ-MSCs’ secretome triggers neuronal survival and differentiation in vitro and in vivo [73, 74]. Secreted factors such as VEGF-A, angiopoietin-1, fibroblast growth factor (FGF)-I, hepatocyte growth factor (HGF), FGF-II, brain-derived neurotrophic factor (BDNF), glial cell line-derived neurotrophic factor (GDNF), and platelet-derived growth factor (PDGF)-AB were identified [75–79]. Importantly, MSCs’ secretome alters both adaptive and innate immune responses [55, 80]. MSCs inhibit autoreactive T cell responses in animal models of multiple sclerosis and hypoxic-ischemic brain injury [81–83]. MSCs shift the alveolar macrophages from a M1 (pro-inflammatory) to a M2 (protective) phenotype ameliorating pulmonary injury in acute LPS-induced acute lung injury model [84]. Thus, the shifting from the M1 to the M2 states and promoting regulatory T cells are a function unique to MSCs [85]. Besides T cell modulation, MSCs inhibit B cell proliferation, neutrophil and monocyte function, and NK toxicity [86–89]. Although these modulatory effects are partially understood, direct cell-to-cell contact and soluble factors are relevant [90]. Additionally, MSC effects expand beyond constitutive immune modulatory properties with the release of cytokines and growth factors such as VEGF, transforming growth factor beta-1 (TGF-β1), TNF-α, interleukin-1 (IL-1), interleukin-6 (IL-6), and IFN-γ [91–94].

## MSCs: regeneration/replacement of injured cells

MSCs’ multipotency and self-renewal properties make them valid candidates for providing both lung and brain cell regeneration/replacement. Although, this strategy for repair carries risks such as tumorigenic potential [37, 38], MSCs were successfully differentiated into various types of cells including cardiomyocytes, myocytes, and epidermal and endothelial cells [54, 95–97]. Importantly, MSCs express neuroglial commitment and can be differentiated into lung cells as well [98–102]. Not surprisingly, MSCs are currently tested in various animal models and clinical trials for lung and brain regeneration [103–105]. Although this line of investigation is particularly intriguing, MSCs’ potential to replace injured cells is not proven and is a matter of constant debate [103, 104, 106]. For example, intravenously injected MSCs improve myocardial infarction without permanent replacement of injured cells [107]. In the lung, MSCs embolize causing endothelial damage and are cleared in a matter of hours [107]. Taken together, the MSCs’ low rate of in vivo engraftment and differentiation suggests that transplanted cells affect tissue injury and repair through paracrine factors. Whether the factors released by the MSCs or the cells themselves are more promising for the therapy of lung and brain injury in the newborn still remains an open question.

## MSCs: extracellular vehicles

The translation from bench to bedside requires the most efficacious and safest approach. Thus, the question of cell-based versus cell-free therapy needs to be addressed. Given that the MSCs’ therapeutic potential has been shown to be largely triggered via paracrine effects and not differentiation, recent studies focus on extracellular vehicles (EV) [108]. These are all types of vehicles present in the extracellular space, including shedding vesicles, apoptotic bodies, and exosomes. Exosomes (40–100 nm in diameter) are secreted by cells in a regulated fashion, possess the ability to transfer proteins and functional genetic materials such as mRNA and microRNAs, and are involved in cell-to-cell signaling and regulation [80]. Not surprisingly, MSC-derived EV are contributing to tissue repair in brain injury including stroke and Alzheimer’s disease [109–111]. The cell-free approach is very promising; however, it is still in its infancy. In fact, stem cells do not just secrete growth factors and/or cytokines but encourage the growth and even supplement (host) cells [112–114]. Importantly, the stem cells’ potential of immunomodulation and protection after injury seems to depend on the bidirectional communication between the injured host cells and the graft via the exchange of specific information [115].

## MSCs: clinical trials

As a result of the remarkable regenerative potential of MSCs, MSCs are ideal candidates for clinical cell therapy. MSCs are easily available, have a good safety profile and homing capacity, and importantly are relatively immunoprivileged, allowing allogeneic transplantation. Not surprisingly, MSCs have been tested in clinical trials in several neurodegenerative diseases such as stroke [116–118], amyotrophic lateral sclerosis [119], multiple sclerosis [120, 121], and spinal cord injury [122]. Several clinical trials indicated no serious side effects or dose-limiting toxicity in acute respiratory distress syndrome [123] and chronic obstructive pulmonary disease [124, 125]. Also, safety and feasibility trials for CP [126–128] and BPD [129, 130] were successful. A recent double-blind randomized control study used allogeneic umbilical cord blood, in combination with erythropoietin, in children diagnosed with CP showing motor and cognitive benefits [131]. In contrast to previous studies, a large safety study used autologous umbilical cord blood directly after birth for infants at risk for hypoxic-ischemic encephalopathy [132]. This approach differs significantly from previous studies as it aims mainly to prevent and not to replace affected cells. Cells were transplanted directly after birth and in combination with hypothermia. Authors concluded that the collection, preparation, and infusion of fresh autologous umbilical cord blood cells for use in infants with hypoxic-ischemic encephalopathy are feasible. The feasibility and safety of intra-tracheal infusion of umbilical cord-derived MSCs in BPD was reported as well [129]. MSCs reduced BPD severity and retinopathy of prematurity and improved inflammatory cytokine profile in tracheal aspirates in a phase 1 dose escalation study. Several other clinical trials for neonatal brain and lung injury are currently listed as in progress or completed, and more results should become available in the near future (ClinicalTrials.gov Identifiers: NCT01297205, NCT01828957, NCT02023788, NCT01832454, NCT01962233, NCT01988584, NCT01207869).

## Conclusions

Given the array of potential regenerative mechanisms of MSCs to protect neonatal brain and lungs, therapeutic applications can be envisioned in the near future. The mechanisms span anti-apoptotic/pro-mitotic capacities leading to neovascularization by the stimulation of angiogenesis and anti-inflammatory responses. Further, stimulation of neuro- and gliogenesis, synaptogenesis, neurite outgrowth, and also immunomodulation are crucial. Available data clearly demonstrate that MSC and secreted factors are beneficial to treat a variety of neurodegenerative and lung disorders. Although the data are very promising, critical questions need to be answered:

- First, what are the appropriate age, passage, and dosage of the transplanted MSCs? Freshly isolated MSCs may be beneficial compared to cultured cells, but is this practicable in a clinical setting especially in the context of preterm infants [133]?
- What is the best source and application route of MSCs? Banking of UCB and placental tissue offer an easily accessible and ethically responsible source of MSCs, and minimally invasive routes are currently tested [47, 66, 134–136].
- Finally, how does the transplantation of MSCs impact the standard care in the neonatal intensive care unit? The lack of effective interventions for many morbidities related to prematurity unlocks the potential of cell-based personalized treatments. Safe and effective clinical interventions are future perspectives bearing hope to improve the lifelong outcomes of the infants in our care. This has to be proven in long-term follow-up studies.
